# An Overview on Causes of Nonadherence in the Treatment of Rheumatoid Arthritis: Its Effect on Mortality and Ways to Improve Adherence

**DOI:** 10.7759/cureus.24520

**Published:** 2022-04-27

**Authors:** Tutul Chowdhury, Jui Dutta, Pharlin Noel, Ratul Islam, Gael Gonzalez-Peltier, Samzorna Azad, Malavika Shankar, Aditya Keerthi Rayapureddy, Padmaja Deb Roy, Nicole Gousy, Khondokar N Hassan

**Affiliations:** 1 Internal Medicine, One Brooklyn Health System, Brooklyn, USA; 2 Medicine, Comilla Medical College, New York City, USA; 3 Surgery, Mount Sinai South Nassau Hospital, Oceanside, USA; 4 Medicine, American University of Antigua, New York City, USA; 5 Internal Medicine, One Brooklyn Health System, New York City, USA; 6 Medicine, Bangladesh Medical College and Hospital, Dhaka, BGD

**Keywords:** socio-economic factors, co morbidity, non-adherence, drug efficacy, dmards, chronic joint pain, rheumatoid arthritis

## Abstract

Rheumatoid arthritis is one of the most prevalent musculoskeletal disorders that, when insufficiently treated, results in detrimental sequelae including joint damage and reduced quality of life. Poor patient adherence to medication is a significant blockade to effective management. The purpose of this review is to highlight and discuss the factors responsible for defiance of antirheumatic medication and ways to overcome these barriers. Education level, health literacy, cohabitation status, multi-morbidities, complicated drug regimen, intermittent co-payments, prescribed regimen adverse effects, and cognitive impairment are a few among many common barrier factors leading to poorer outcomes in rheumatoid arthritis. While there is an abundance of inhibitory factors leading to worsening disease progression, they each can be easily dealt with an effective approach at the beginning or during the treatment course to ensure a better outcome.

## Introduction and background

Rheumatoid arthritis (RA) is a chronic systemic autoimmune inflammatory arthritis most directly affecting the synovial joints in addition to other organs including the heart and lungs [1]. RA affects approximately 1% of the population and leads to chronic pain, fatigue, increased risk of cardiovascular diseases, infection, and premature death [1,2]. The global age-standardized prevalence rate has increased 7.4% to 246.6/100,000 in 2017, with an additional increase of RA incidence to 14.9 since the 1990s and is predicted to increase further still [2]. It has also been reported by the Global Burden of Diseases, Injuries and Risk factors study that age-standardized prevalence and disability-adjusted life years rates were increased in females and those older than 70 years of age [2]. With increased expected life spans, RA is becoming an increasingly prevalent issue in the patient population. Incompletely managed RA leads to significant joint degeneration, functional impairment, morbidity, and an increased risk of death [1]. 

Therapies for RA include analgesics, NSAIDS, glucocorticoids, and disease-modifying antirheumatic medications (DMARDs) [3]. DMARDs reduce disease activity and radiographic progression while also improving long-term functional outcomes [4]. The use of a conventional DMARD-methotrexate (MTX), sulfasalazine, an antimalarial agent, or leflunomide-as first-line medication is frequently recommended by institutions worldwide [3]. Optimal MTX use has a number of advantages including disease management, a reduction in the need for more expensive biologic treatments, and an improvement in the patient's overall outcome [5]. Unfortunately, adherence to treatment is low in individuals with RA, ranging from 30% to 80% [6]. Nonadherence to DMARDs in patients with RA may result in unnecessarily high levels of disease activity and loss of function in the affected joints [4]. The World Health Organization (WHO) categorizes factors relating to nonadherence into five categories; socioeconomic factors, healthcare system factors, condition-related factors, therapy-related factors, and patient-related factors [6].

Medical nonadherence is either characterized as intentional or unintentional [6]. We can categorize intentional sources for non-adherence as those where the patient chose to skip a medication dosage or cease taking medication based on their own decision without consulting their physician. This can be based on the patient’s beliefs, based on adverse effects (AEs) they may be experiencing, or due to limited improvement of their symptoms. These factors weigh into a risk-benefit analysis that patients make based on their goals of treatment, their conceived notions about their medications, and their knowledge of their disease [6]. According to the Self-regulatory Common Sense Model of illness and treatment, how a patient perceives their illness and how effective the treatment is will play a significant role in patient medication adherence [7]. On the other hand, unintentional adherence is characterized by factors outside of the control of the patient. This can include medication costs, regimen complexity, or patient forgetfulness [6]. It is critical to determine whether or not there are other variables or barriers contributing to nonadherence and to elucidate interventions that may enhance adherence and improve outcomes.

Several studies have attempted to investigate barriers to drug adherence in RA patients. Our research will review the literature to determine what factors contribute to patients with RA failing to adhere to their treatment regimen, the effect of nonadherence on mortality, and how to improve adherence. We sought to determine the impacts of how several seemingly unrelated factors can create significant obstacles in patient medication adherence.

Methodology

**Figure 1:**
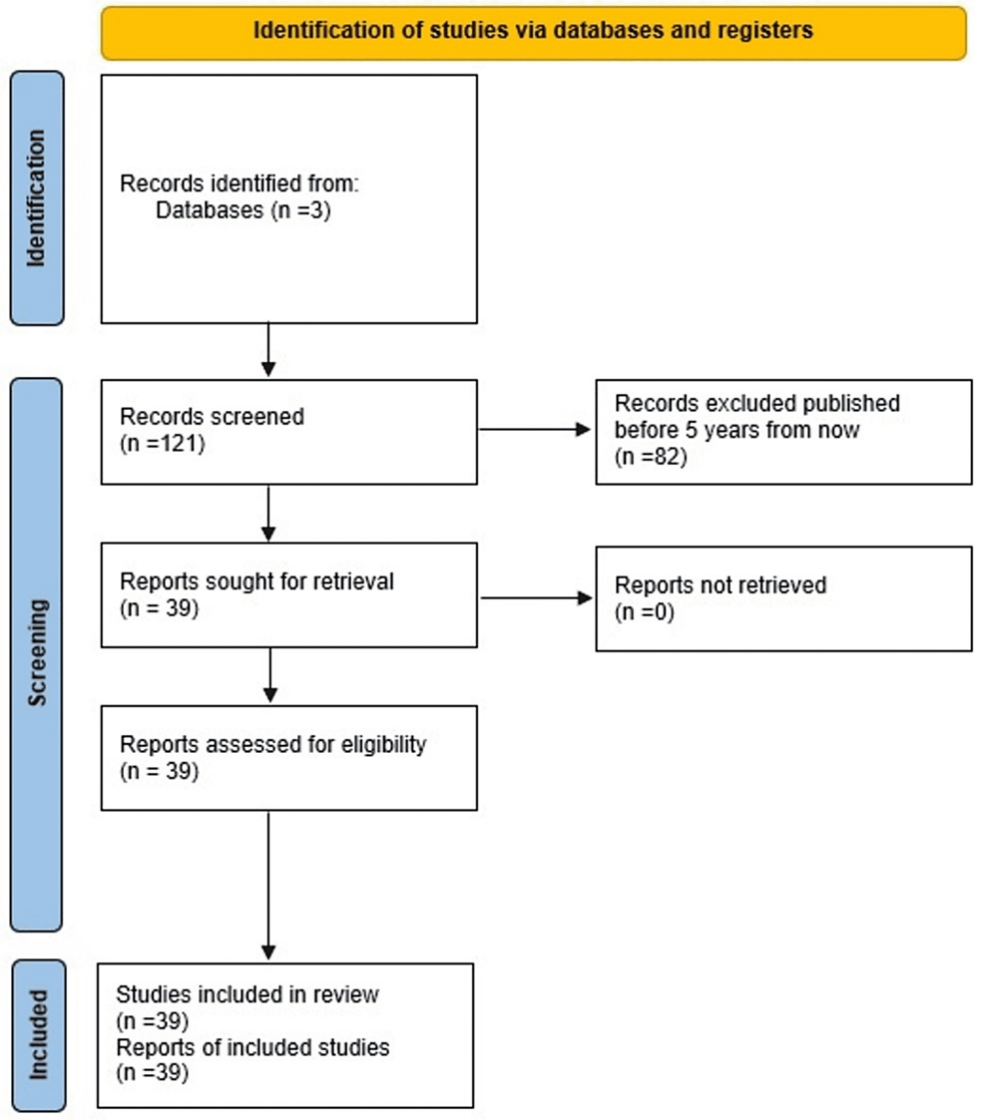
Screening Flowchart: Preferred reporting items for systematic reviews and meta-analyses (PRISMA) flow diagram showing the study selection process

Articles and studies addressing effective treatment adherence, and key factors contributing to nonadherence were identified by effective keywords search in Pubmed central, Google Scholar, Cochrane, as well as the references found within these articles. Data collection and extraction were performed and articles were further scrutinized to maintain methodological quality (Figure 1).

## Review

Socioeconomic factors and their impact on therapy adherence

Socio-economic status is a crucial component that determines adherence to prescribed treatment in chronic diseases like RA. Association of high out-of-pocket costs with non-adherence to treatment was also reported widely [1,8-11]. Curkendall et al. showed that patients with RA having out-of-pocket costs above $50/week dropped follow-up visits by 25% compared to patients with out-of-pockets below $50/week [11]. Another study found that nearly 70% of the patients cannot comply with prescribed medication due to their tight financial conditions [12]. Insurance coverage also influences a patient’s decision to take or reject medication [10]. Patients’ inability to afford medication leads to uncontrolled prognosis with associated complications like disability and may require them to operate on joints [11].

The effect of the healthcare team and system-related factors on adherence 

Health system-related factors are involved in every aspect of the physician-patient relationship and can influence the effectiveness of a therapy regimen. Studies have shown that system-related factors such as patient education, the physician-patient relationship, the available and accessible information about a particular disease, or even potential drug supply shortages can all variably influence non-adherence to medication [13]. One example of this is how medications can be introduced to patients by their physician. Non-adherence with medication, in some cases, is actually triggered by a patient's inadequate awareness of the reason a new drug is being added, and what they may expect when taking a new medication. One study showed that 65% of the non-adherence associated with initiating MTX occurred because patients were not aware of its actual usage or why they were taking the medication [3]. This necessitates the importance of appropriate and sufficient patient education, especially when creating treatment plans that require the use of medications. 

The use of visual tools has proven to be effective in orienting and educating RA patients in several studies [14]. One such technique is the use of musculoskeletal ultrasound through which patients were able to view “real-time” images of their own joint anatomy and see the progression of their disease process and the consequences of joint inflammation in real-time [1,15]. This allowed patients to interact with their physician with a better working knowledge of their disease process. Studies have also shown that patients' engagement was higher when interactive visual tools are used [1]. This could be due to making their disease progress less theoretical and more practical. The use of these visual tools additionally provides a platform for the physician-patient relationship to develop, and for deeper discussion to ensue. The use of visual tools is a simple way to overcome some of these barriers involved with patient education to improve medication adherence.

Psychiatric-related factors on therapy adherence 

Adherence to medication is greatly affected by psychological irregularities. Several studies have shown an association between medication adherence and mental conditions [13]. Major depressive disorder and anxiety are the two major psychological irregularities frequently reported in association with medical non-adherence and RA [16-18] Major depressive disorder in particular involve a wide variety of symptoms including pain, tender joints, disability, sleep disturbance, and increased rates of early retirement [16-18]. A study investigated the effect depression has on medication non-adherence which revealed that patients suffering from depression reported a 2.3-fold increased rate of medication non-adherence than non-depressed patients [13]. While there is a potential association of major depressive disorder with the development of inflammatory cytokines, the psychological toll of depression on a patient’s ability to remember to take the medication, and their drive to take the medication are serious obstacles to the goal of medication adherence that should be considered by the physician when creating a patient treatment plan.

Therapy-related factors

The complexity of a patient’s drug regimen, which includes but is not limited to multiple medications, numerous doses, and specific time requirements, is related to poorer adherence to RA [19,20]. A review of literature studies was analyzed, which confirmed that the number of doses per day, as well as the complexity of the drug schedule, was inversely proportional to adherence [6]. Less frequent dosing resulted in better adherence across a variety of therapeutic classes. Intentional non-adherence is affected by the patient’s belief of perceived side effects, perceived benefits of a certain drug regimen, and degree of understanding of therapeutic benefits [6]. In a cross-sectional study using 122 patients diagnosed with RA, the common reason for MTX non-adherence in RA treatment had been a lack of understanding of the slow-acting potential of MTX [21]. The patients did not see immediate benefits of MTX to treat their stiffness and joint pain in the first few weeks, which displayed a lack of understanding of the drug’s time to therapeutic action. Patients had lost confidence since they resented the wait for weeks due to the slow-acting nature of DMARDs. It seemed unfamiliar compared to fast-acting drugs such as corticosteroids, which are used on a simpler “by need” basis.

Route of administration is another obstacle to adherence. Oral MTX formulation is associated with two times increased risk of non-adherence compared to the parenteral route [22]. In a study conducted, the non-adherence to MTX treatment was observed in 38% of patients (n=1108) with oral MTX with a high risk of non-adherence (Hazard ratio-HR 2.11, 95% confidence interval-CI 1.87-2.39, p < 0.001) [22]. For parenteral route of administration of MTX with doses of 7.5,10, and 15 mg weekly doses, there had been lower risk of non-adherence (HR 0.79, 95% CI 0.63-0.99, p = 0.038; HR 0.62, 95% CI 0.55-0.71, p < 0.001; HR 0.7, 95% CI 0.58-0.85, p < 0.001, respectively) [22]. In another study involving 207 patients, switching from oral to subcutaneous route increased the proportion of remission from 22.8% to 52.9% [23]. The subcutaneous route increases MTX bioavailability whereas the oral MTX bioavailability reached a plateau, regardless of dose, due to gut absorption. There were increased intracellular long-chain MTX polyglutamates which were associated with a better clinical response and improvement in adherence [23].

Another reason for nonadherence to therapy is due to side effects. The Rheumatoid Arthritis Medication Study (RAMS), a prospective multicenter cohort study, was conducted involving 606 patients with RA, who were tracked within the first six months of MTX initiation for predictors of non-adherence [5]. Of those who were non-adherent, 112 (71%) were intentionally and 31 (19%) were non-intentionally non-adherents, the main reported reason for not taking MTX included MTX side effects (34%). Side effects were reasons for non-adherence (n=53) which included nausea (74%), fatigue (45%), headache (30%), and dizziness (26%) [5]. At one year, up to half of RA patients discontinue MTX use, leading to substandard adherence, to avoid side effects [24]. Adverse reactions to drugs, fear of infections, and also anxiety due to health concerns play a large factor in nonadherence to therapy.

Medication AEs

AEs of medications are one of the highest sources of anxiety and potential nonadherence for patients with RA [6,25-27]. The influential power of preconceived AEs of a medication can induce medication nonadherence before actual perceived AEs can be reported [27]. In a study on how patients generally view medications, specifically biologic agents used in the treatment of RA, it was revealed that overall, the patients interviewed believed that all medications are harmful in the long run and that side effects were inevitable, despite their knowledge of how effective they are [27]. This is in concordance with several other studies where the anxiety associated with possible AEs is a significant and primary source of nonadherence [26]. 

An additional factor to be considered in the development of AEs is the treatment for other comorbid diseases in addition to RA. When multiple medications are prescribed, the risk of medication interactions increases along with the risk of AEs [28]. One study saw that when MTX was combined with frequent corticosteroid use, tolerance to MTX dropped significantly (odds ratio = 2.73; 95% confidence interval, 1.06 to 7.06; p = 0.038) [29]. When AEs become too severe or frequent, the patient-perceived risk-benefit declines leading to skipped doses or abandonment of the medication altogether [5,29]. Even chronic fatigue, a common AE reported from those on multi-pharmacologic treatment regimen, was reported to cause such a detrimental emotional impact that eventually lead to nonadherence [5,28].

There also appears to be a disconnect between AEs perceived by physicians versus AEs experienced by patients. On average, rheumatologists estimated an average rate of AEs of 15% for patients receiving MTX and leflunomide, and a rate of merely 10% in those receiving sulfasalazine and hydroxychloroquine [26]. However, the same study revealed that of these patients approximately 40% were experiencing AEs with MTX, 33% reported AEs after taking leflunomide, and approximately 49% reported AEs after taking sulfasalazine, and approximately 15% reported AEs after taking hydroxychloroquine [26].

This large gap also extends to what AEs are identified in physicians versus those identified by patients. The most commonly reported AEs by physicians were those with respect to laboratory and imaging results, such as cytopenias, interstitial lung diseases, and liver or kidney function impairment [26]. Additional research showed that when taking MTX, one of the most common medications used in the treatment of RA, nausea was experienced by 92.3% of patients, followed by irritability and restlessness (96.1%), abdominal pain (46.1%), and vomiting (30.7%) [27,29]. However, with these side effects in mind, patients were hesitant to report them for fear of seeming “difficult” or receiving negative feedback from the physician [27]. This fear is additionally exacerbated by shorter consultation times common in everyday practice [25]. One way to address this issue of the disconnect between patient and physician is to allot more time for the patient to express their concerns during consultations while additionally creating an open space that allows for an honest discussion regarding AEs [5,27]. 

Patient-related factors

A number of patient-related factors have been found to influence medication adherence in RA patients. In a prospective cohort study involving 443 adult, Thai RA patients Katchamart et al. found that age and functional status as measured by the Health Assessment Questionnaire (HAQ) were significantly related to medication nonadherence [3]. Older age and more functional impairment significantly decreased the risk of nonadherence (age (risk ratio, 0.98; 95% CI, 0.96-0.99; p, 0.048) and HAQ (risk ratio, 0.62; 95% CI, 0.39-0.98; p, 0.041)). A Polish cross-sectional study in which the median age of subjects was 58 years old yielded similar results. Bak-Sosnowska et al. concluded that elderly patients appeared to use pharmacotherapy more systematically, which may result from a greater level of adaptation to the disease and its acceptance compared to younger patients, but also from objective factors, such as regular daily routine or more free time [2]. On the other hand, through ANOVA analysis, age was not identified as a predictor of adherence in a cross-sectional, multi-center study performed in Spain which is consistent with results obtained by Alrubaye et al. in Iraq [20,21].

Another contributory factor to medication nonadherence in RA patients is cohabitation status. Pombo-Suarez et al. found that in a multivariate analysis adjusted for age and sex, cohabitation was independently associated with adherence in the Spanish population [20]. Social and family support seem to be important factors contributing to proper adherence and living alone has been previously associated with poorer adherence. 

Education level and health literacy are important considerations regarding medication adherence in RA patients specifically. In a recent Iraqi study that assessed the causes of non-adherence to MTX in patients with RA, the only factor that affected the adherence of patients significantly was education level. The other sociodemographic factors considered in the study (ie. age, gender, and socioeconomic status) had no effect on MTX adherence. Patients with lower levels of education showed better adherence than the higher educated groups. It was suggested that these results may have occurred as a result of the higher educated patients’ ability to use the internet in seeking answers about the long-term side effects of the drug [21]. Health literacy refers to the ability to acquire, comprehend, and pursue health information to guide health-related decisions [1]. Low health literacy impairs patients ability to comprehend medication labels, names, and instructions. A practical suggestion is that clinicians should use visual tools such as videos and pictorial aids to assist in the meaningful delivery of key health messages [1].

Cognitive impairment on therapy adherence

During the height of the COVID-19 pandemic, many patients experienced a worsening of their symptoms in addition to significant emotional and mood disturbances attributed to prolonged self-isolation [30]. One patient survey revealed approximately 20% of patients intentionally ceased taking their medications during this period despite having tolerated the medication prior. While the worsening of symptoms may be in part due to restricted availability for daily physical activity, it may also be due to an increase in anxiety and sadness provoked by prolonged confinement or fear related to contracting the COVID-19 virus [30]. Additionally, worsened pain and joint stiffness can also directly increase anxiety and patient-reported sadness, potentially leading to medication nonadherence. In a study of 644 patients, 63% of those who intentionally stopped their treatment did so out of fear of developing COVID-19, and 49% of these patients did so without consulting their physician [30]. While the relationship between the psychological effects of prolonged self-isolation and the increased societal anxiety regarding the pandemic may not have a clear relationship to medication nonadherence, more studies need to be done to explore how depression and anxiety can affect patients and their RA treatment.

Depression can drastically alter planning ability, memory, and beliefs about therapy and efficacy [31]. One of the most common unintentional causes of nonadherence is attributed to a patient’s forgetfulness in taking medication [32]. This could simply be attributed to the failure to form a habit of taking medication, however, the higher rates of depression in patients with RA might have a contributed to patient forgetfulness [18,31]. Major depressive disorder and anxiety have a wide variety of causative factors, these include pain, chronic inflammation, and physical disabilities, however, studies have shown a direct link between chronically elevated inflammatory cytokines and the development of major depressive disorder [18,31]. Pro-inflammatory markers, such as interleukin (IL)-1, IL-6, tumor necrosis factor-alpha, and C-reactive protein, have been shown to be significantly elevated in those diagnosed solely with the major depressive disorder as compared to those without. These pro-inflammatory markers could potentially induce neuroinflammation and lead to the forgetfulness, sadness, and poor executive functioning seen in patients with depression and other chronic diseases [31]. 

Several studies saw that depression had a notable impact on medication adherence. In a study about nonadherence in rheumatoid patients using DMARDs, Xia et al. found depression to be negatively associated with medication adherence, in addition to patient education, income, and the total number of DMARDs used [33]. Another study, in particular, saw that those with depression were more forgetful in taking their medications than those without (Pearson coefficient = 0.317, p<0.01) [31]. Whether the depression stems from inflammation, or from a patient’s inability to cope with their disease, major depressive disorder is undoubtedly a significant factor in patient medication adherence. Particularly, medication adherence can be a symptom of worsening major depressive disorder, especially in those with chronic inflammatory diseases.

External forces on therapy adherence

After interviewing 228 patients with RA who were taking DMARDs and completed self-report questionnaires such as the Compliance Questionnaire on Rheumatology (CQR) and the Medication Adherence Scale (MARS) to assess adherence, Bart et al. concluded that only three variables were associated with adherence in their study: disease duration, the number of medication-related adverse events, and beliefs about the medication's necessity [4]. All three of these variables have the potential to impact external forces on therapy adherence. In terms of patient beliefs about medications necessity, patients with any nonadherence behavior had lower overall and necessity scores on the Beliefs About Medicines Questionnaire (BMQ) than adherent patients [32]. Professor Robert Horne and colleagues developed the BMQ as a way of examining cognitive representations of medication, and it has been validated for use in patients with common chronic conditions and non-adherence has a negative correlation with the overall BMQ score [34]. Patients' beliefs about their sickness and medications are associated with early nonadherence [5]. Patient beliefs in medicines and their experience in controlling their illness were both found to be linked to non-intentional non-adherence [32]. As a result, quick interventions that address patient concerns while increasing understanding of the medication's necessity may enhance adherence. In a recent prospective study of 443 patients with RA followed for a year and a half, nonadherence was found to be prevalent at 22% [3]. In this study, the most common reason for nonadherence was forgetting to take medications due to a hectic work schedule (51%), followed by running errands (26%), and complicated drug regimens (16%) [3]. Most common non-intentional reasons for nonadherence, such as forgetting or running out of medication, have been documented in other studies [5,32]. Non-intentional nonadherence behaviors are found to be associated with younger age, the requirement for more medicine dosages per day, and an inverse relationship with the BMQ overall score [32]. Co-morbidity is also a significant predictor of nonadherence [32]. Multi-morbidity increases and complicates the health-management task confronting patients, thus increasing the likelihood of running out of or forgetting prescriptions and decreasing non-intentional adherence to medication [5].

Medication adherence improvement strategies 

**Figure 2:**
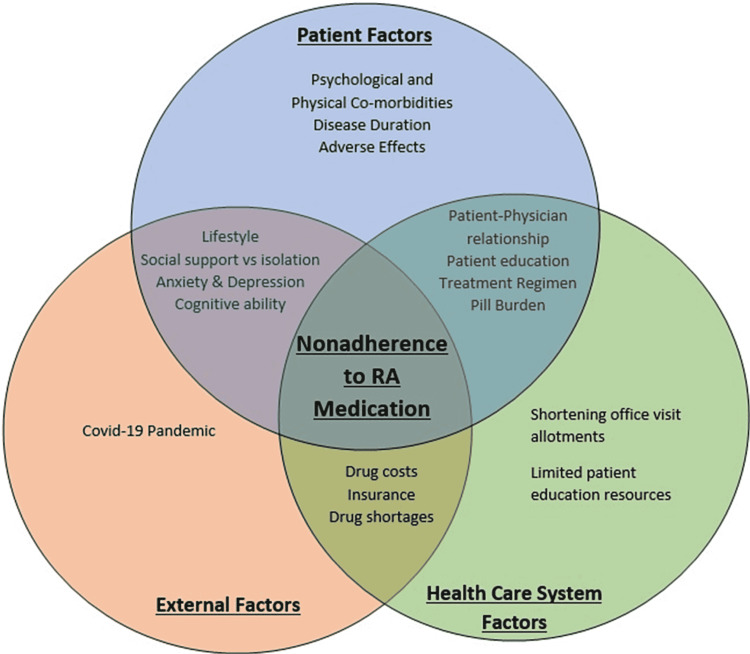
A schematic representation of the factors involved in medication nonadherence in rheumatoid arthritis and how they overlap

Interventions to improve medication adherence do exist and strategies have been proven to better patient medication-taking behavior. Understanding the confounding factors reducing patient medication adherence is by far one of the biggest steps toward improving adherence (Figure 2) [35]. This can be split into patient-related factors and physician/system-related factors, however, regardless of categorization, understanding these obstacles is essential in creating systems to improve patient medication adherence [35]. A major way to overcome these hurdles is with improved patient education. Effective patient education using differing modalities tailored to each patient can help dissipate confusion associated with the disease and treatment modalities, ease the anxiety associated with medication use, and provide a platform to encourage a discussion regarding the patient's beliefs and goals of treatment [36,37]. It was recently reported that approximately 40%-60% of patients were not able to correctly recall medication instructions verbalized to them by their physician [38]. Another study revealed that over 60% of patients were confused about directions regarding medication schedules after visiting their physician [38]. This indicates that patient education is a serious issue, however, it can be easily remedied. Several studies have been done to evaluate the effectiveness of patient education on adherence to RA medication and many have shown that using various teaching modalities such as visual aids, charts, and allowing for open discussion, have a significant positive impact on increasing medication adherence, and ultimately disease management [1]. This is especially true for patients with lower literacy levels where studies have shown a significant increase in medication adherence when a multi-modality approach to patient education was utilized [37,38]. Overall, improving patient education regardless of literacy level emphasizes disease and treatment understanding, and allows the patient to express their concerns. This can significantly improve adherence while also providing the opportunity to build trust within the patient-physician relationship [39].

Another approach to overcoming medication nonadherence includes simplifying the treatment modality [38]. Understandably, when a treatment regimen is complex, medications are more likely to be improperly timed or administered or missed entirely [38]. This is especially true in patients with limited cognitive ability, or those with limited time or access to medications [37,38]. An effective way to combat this issue is simply by reducing the pill burden. This method has been shown to be effective in the treatment of other chronic diseases such as hypertension or diabetes mellitus [35]. Reducing the medication burden through reducing the number of pills, or simplifying the treatment regimen has significantly improved medication adherence in other diseases, however, the data regarding the effectiveness has not been thoroughly investigated in the literature to date [37].

## Conclusions

A multitude of factors influences medication nonadherence in patients with RA. These factors center around socioeconomic constraints, the effects of the healthcare team, and condition-related factors like depression. Therapy-related factors including the complexity of the drug regimen and route of administration as well as medication adverse effects also play important roles. Patient-related factors such as age and education level appear to be significant in some but not all patient populations. Depression causing cognitive impairment was identified as a major deterrent to therapy adherence, particularly during the height of the COVID-19 pandemic. Patients’ beliefs about medication necessity, forgetting to take medications due to a hectic work schedule, and co-morbidities were all identified as external influences on medication adherence.

Preventing disease progression while maintaining a low treatment burden for patients with RA can be quite a challenging balance to achieve. This makes understanding the various reasons for patient nonadherence even more crucial to keep in mind when creating a treatment regimen. When this balance is not achieved, suboptimal management can unnecessarily provoke disease progression, which can lead to worsening joint symptoms for patients, ultimately reducing their ability to perform activities of daily living. While this review provides an updated look at the underlying factors for patient nonadherence, there is little in the literature addressing the consequences of suboptimal RA therapy.

In order to improve medication adherence in RA patients, a number of strategies have been explored such as implementing patient education, allowing patients to understand the disease, and how their medication regimen can easily halt progression. Many studies have explored various ways that patients can be educated, as comprehension skills and literacy levels vary among patients. Information delivery that includes visual elements such as imaging of joints affected by RA has proven to be effective. Patient education is also crucial as many patients are afraid of medication adverse effects and unable to comprehend how the benefits outweigh the negative consequences.

Improving physician-patient relationship dynamics, specific factors such as trust and communication skills have also been proven to be effective. The patient population is diverse, including individuals from different backgrounds and literacy levels, therefore, a disconnect often develops between providers and patients, and/or patients may feel that their provider is imposing medical control. Such situations affect medication adherence as patients are not able to understand their provider, their disease, or their disease management. Furthermore, cognitive-behavioral interventions, modifying medication type and regimens centered on individual patient satisfaction, and improving the socioeconomic aspect of healthcare to benefit patients of all financial situations, can all increase medication adherence.
